# Severe Juvenile Dermatomyositis With Peripheral Nervous System Involvement: A Case Report

**DOI:** 10.7759/cureus.103707

**Published:** 2026-02-16

**Authors:** Víctor M Mora-Bautista, Jenifer Walteros-Cárdenas

**Affiliations:** 1 Pediatrics, Universidad Industrial de Santander, Bucaramanga, COL; 2 Pediatrics, Clínica San Luis, Bucaramanga, COL; 3 Pediatrics, Universidad de Santander, Bucaramanga, COL

**Keywords:** child preschool, dermatomyositis, dermatomyositis juvenile, peripheral nervous system diseases, polyneuropathy

## Abstract

Juvenile dermatomyositis is the most common inflammatory myopathy of childhood. While many cases achieve favorable outcomes, severe forms may develop persistent disease activity, complications, and long-term disability.

A five-year-old girl was admitted after 20 days of Gottron's papules and heliotrope rash, with intermittent fever during the first 10 days. She then developed mild proximal-predominant muscle weakness in all four limbs, rapidly progressing to severe generalized weakness with signs of axonal neuropathy over the subsequent 15 days. Despite aggressive immunosuppression with IV methotrexate, high-dose methylprednisolone pulses, IV immunoglobulin, oral prednisolone, and azathioprine, she demonstrated inadequate clinical response over four years of follow-up.

Severe juvenile dermatomyositis with peripheral nervous system involvement may follow a refractory course despite guideline-directed aggressive immunosuppression. This case highlights the therapeutic challenges of rapidly progressive disease with atypical neurological complications and underscores the need for early recognition and intensified treatment strategies in high-risk presentations.

## Introduction

Juvenile dermatomyositis (JDM) is the most common autoimmune myopathy of childhood, with an incidence of three cases per million population, a female-to-male ratio of 2:1, and a bimodal age of onset (5 and 11 years). It is characterized by symmetric proximal muscle weakness, characteristic cutaneous rash (Gottron's sign, heliotrope rash, and malar erythema), elevated muscle enzymes, electromyography (EMG) findings of myopathy, and typical muscle biopsy [1]. It represents an inflammatory and endothelial cytopathic occlusive vasculopathy affecting muscles, skin, heart, gastrointestinal tract, and central nervous system (CNS)/peripheral nervous system (PNS) [2]. Diagnosis relies on the 2017 American College of Rheumatology (ACR)/European League Against Rheumatism (EULAR) criteria, incorporating biopsy and myositis-specific autoantibodies [3,4], updated in 2020 to include magnetic resonance imaging data [5].

This disease has a favorable prognosis in 50% of cases, with improvement after one to two years of treatment. The remaining 50% includes severe and refractory cases with complications such as calcinosis, gastrointestinal involvement, pulmonary disease, prostration, and even fatalities [4,6]. JDM-associated peripheral neuropathy is exceptionally rare (<1% cases) [1], often portending severe, treatment-resistant disease. This paucity of reports underscores diagnostic and therapeutic challenges.

We present a severe, rapidly progressive case due to its atypical presentation, diagnostic challenge, and poor prognosis.

## Case presentation

**Figure 1:**
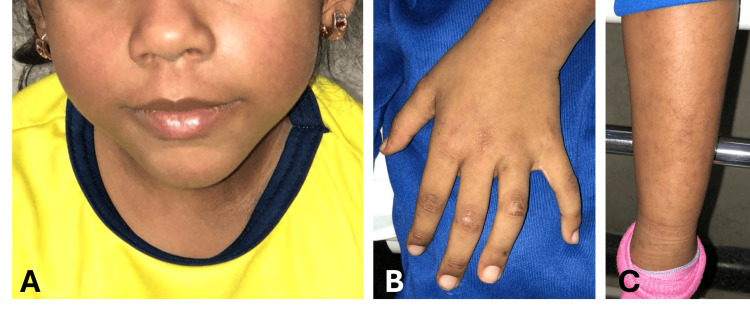
Cutaneous manifestations in the reported case (A) Facial exanthema and edema. (B) Gottron papules over the interphalangeal joints. (C) Erythematous hyperpigmented macules in the left pretibial region

A five-year-old girl presented to the emergency department with erythematous hyperpigmented macules on the extensor surfaces of elbows, knees, and interphalangeal joints, mild nail bed erythema, and facial edema and erythema of 20 days' duration (Figures 1A-1C).

She also had 10 days of intermittent, low-grade fever (below 39°C) that became daily in the last five days, along with proximal-predominant muscle weakness in all four limbs that progressed to inability to stand (positive Gowers' sign) and severe generalized osteomyalgias. Initial laboratory findings are summarized in Table 1.

**Table 1:** Initial laboratory findings with pediatric reference ranges

Test	Result	Reference range (pediatric)
White blood cells (WBCs)	6,060/µL	5,000–14,500/µL
Neutrophils	28% ↓ (1,697/µL)	30%–60% (1,500–8,700/µL)
Lymphocytes	72% ↑ (4,363/µL)	30%–60% (1,500–6,900/µL)
Hemoglobin (HGB)	11.8 g/dL	11.5–14.5 g/dL
Hematocrit (HCT)	37.50%	34%–40%
Platelets	378,000/µL	150,000–450,000/µL
Erythrocyte sedimentation rate (ESR)	11 mm/h	<20 mm/h
C-reactive protein (CRP)	<6 mg/L	<8 mg/L
Prothrombin time (PT)	14.5 s	11–15 s
Partial thromboplastin time (PTT)	21.6 s ↓	25–35 s
International normalized ratio (INR)	1.04	0.8–1.2
Blood urea nitrogen (BUN)	13.5 mg/dL	7–20 mg/dL
Creatinine	0.34 mg/dL	0.3–0.7 mg/dL
Total bilirubin	0.18 mg/dL	<1.0 mg/dL
Direct bilirubin	0.09 mg/dL	<0.3 mg/dL
Indirect bilirubin	0.09 mg/dL	<0.7 mg/dL
Alanine aminotransferase (ALT)	597 U/L ↑↑↑	8–36 U/L
Aspartate aminotransferase (AST)	1,187 U/L ↑↑↑	15–46 U/L
Total creatine kinase (CK)	30,812 U/L ↑↑↑	5–200 U/L
CK-MB	1,959 U/L ↑↑↑	≤25 U/L
Alkaline phosphatase (ALP)	94 U/L	85–400 U/L
Albumin	3.64 g/dL	3.5–5.0 g/dL
Urinalysis	Non-pathological; amorphous urates +++	No pathological findings

These markedly elevated transaminases and muscle enzymes were initially interpreted as myositis and infectious hepatitis. The differential diagnosis included hepatitis A, leptospirosis, rickettsiosis, parvovirus B19, hepatitis B/C, and typhoid fever; she received empirical antibiotics (ceftriaxone 100 mg/kg/day IV + azithromycin 10 mg/kg/day PO for seven days), discontinued after negative serologies.

Pediatric Rheumatology diagnosed JDM based on Gottron's papules, proximal-predominant muscle weakness (hypotonia, trunk and neck paresis, proximal muscle strength 1/5 and distal 2/5 in all four limbs, deep tendon reflexes (DTRs) +/++++, without notable sensory deficits), and elevated muscle enzymes. EMG and nerve conduction studies revealed poor voluntary recruitment due to weakness, reduced amplitude motor units with rapid recruitment consistent with acute inflammatory myopathy, plus a neuropathic component (mixed axonal and demyelinating sensorimotor polyneuropathy, predominantly axonal, affecting all four limbs but worse in lower extremities), as showed in Table 2.

**Table 2:** Nerve conduction studies of the peroneal nerves Amp, amplitude; CV, conduction velocity; Lat, latency; m/s, meters per second; mV, millivolts; ms, milliseconds. Reference value (RV): Normal reference range The first study showed involvement of the median nerves (motor), ulnar nerves (mixed), peroneal nerves (mixed), posterior tibial nerves (motor), sural nerves (sensory), and saphenous nerves (sensory). The second study showed motor involvement only in the peroneal nerves, the right median nerve, and the tibial nerves.

Nerve	Parameter	Reference value (RV)	First study (diagnosis)	Second study (nine months)
Left peroneal (motor)	Latency	<4.8 ms	4.45 ms	4.30 ms
	Amplitude	>3.0 mV	0.22 mV ↓	0.10 mV ↓↓
	CV	>42 m/s	56.27 m/s	56.00 m/s
Right peroneal (motor)	Latency	<4.8 ms	3.67 ms	2.90 ms
	Amplitude	>3.0 mV	0.44 mV ↓	0.40 mV ↓
	CV	>42 m/s	51.69 m/s	51.00 m/s
Left superficial peroneal (sensory)	Latency	<4.2 ms	8.36 ms ↑↑	7.90 ms ↑↑
	Amplitude	>8.0 μV	0.16 μV ↓↓	0.30 μV ↓↓
	CV	>42 m/s	56.27 m/s	56.00 m/s
Right superficial peroneal (sensory)	Latency	<4.2 ms	8.13 ms ↑↑	6.60 ms ↑↑
	Amplitude	>8.0 μV	0.23 μV ↓↓	0.30 μV ↓↓
	CV	>42 m/s	51.69 m/s	56.00 m/s

Pulse therapy was initiated with IV methylprednisolone 540 mg (30 mg/kg/day) for eight days, with minimal clinical (muscle strength) and laboratory (enzyme levels) improvement. Oral prednisolone 1 mg/kg/day and weekly oral methotrexate 5 mg (7 mg/m² body surface area) were continued. She subsequently developed dysphagia episodes leading to pneumonia treated with parenteral antibiotics. After three weeks of hospitalization with stationary evolution, IV immunoglobulin was prescribed but unavailable through her insurance; she received another course of IV methylprednisolone pulses 30 mg/kg for five days without improvement. She was discharged on prednisolone 1 mg/kg/day and weekly methotrexate 5 mg PO after two additional weeks.

**Figure 2:**
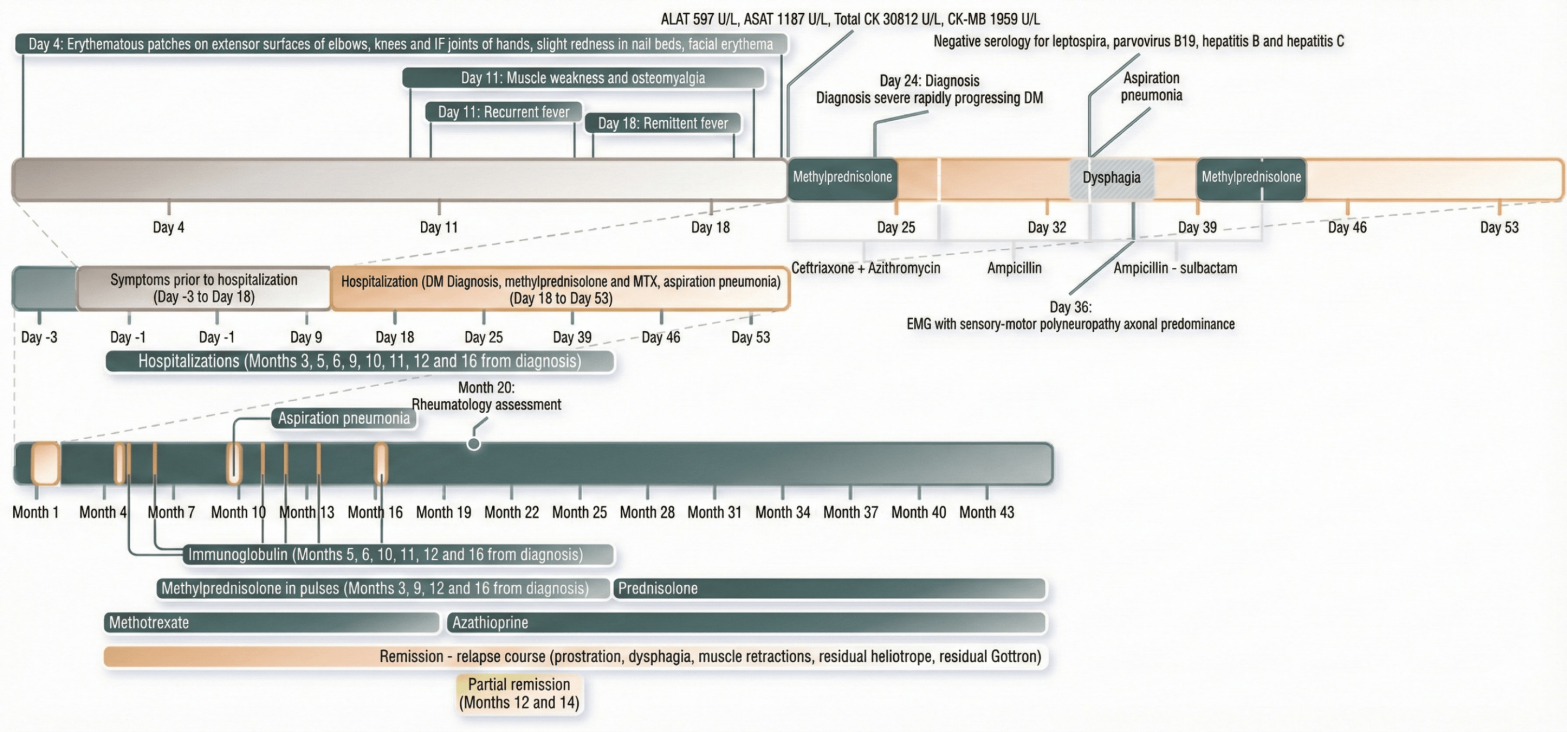
Hospitalizations, treatments, and complications during follow-up DM, dermatomyositis; IF, interphalangeal; MTX, methotrexate.

During 48-month follow-up, treatment course and complications are illustrated in Figure 2. She required additional hospitalizations within the first 16 months for intravenous immunoglobulin (IVIG) infusions (n=7) and received several methylprednisolone pulses (n=6), with ongoing therapies through next months.

EMG at nine months showed severe muscle atrophy without polyneuropathy (Table 2). Methotrexate was switched to azathioprine at 12 months due to lack of improvement. She followed a relapsing-remitting course with partial remissions (Figure 2). Poor psychosocial and economic conditions limited treatment adherence. At final follow-up visit, she exhibited prostration, severe motor or neurological impairment (muscle retractions, strength 1/5, DTRs +/++++, distal hypoesthesia in lower limbs, and bilateral foot drop), vasculitic ulcers, and residual heliotrope rash and Gottron's sign.

## Discussion

We present a definitive case of severe JDM according to the EULAR/ACR criteria for biopsy-negative cases, using their probability calculator for retrospective evaluation (Classification Criteria for Idiopathic Inflammatory Myopathies, EULAR/ACR, http://www.imm.ki.se/biostatistics/calculators/iim/). The patient was classified as high-risk per the SHARE consensus due to severe disability (prostration). Specifically, her Childhood Myositis Assessment Scale (CMAS) score was estimated at 12/52 and her Manual Muscle Testing 8 (MMT8) score at 22/80; these values were retrospectively confirmed based on documented physical examinations showing an inability to maintain posture or overcome gravity at the peak of her illness. Additionally, dysphagia and microaspiration were established early in the disease course [4].​

Regarding diagnostic accuracy, a muscle biopsy could not be obtained due to insurance limitations, and myositis-specific autoantibodies are not readily available locally. However, biopsy is not mandatory for diagnosis (though greater abnormalities predict lower remission probability), and autoantibodies (specific and associated with myositis, positive in 70% of cases) lack a fully established diagnostic value despite phenotype differentiation. Among known autoantibodies, anti-p155/140 and anti-NXP-2 (MJ) are associated with a chronic course and prostration similar to this report [1,4,7].

The hyperacute course was striking, necessitating exclusion of infectious etiologies, with parvovirus B19 infection considered most relevant, given its potential for acute hepatitis and benign myositis [7,8].

Regarding poor outcome predictability, literature identifies risk factors, including male sex, dysphagia (40% of cases, a marker of intensive therapy need), persistence of Gottron's sign beyond three months after diagnosis, and Gowers' sign at diagnosis [9]. This case exhibited all factors except male sex.

PNS involvement evident on EMG was striking and has been termed neuromyositis by some authors. This complication is suspected when JDM or dermatomyositis patients exhibit predominantly axonal motor neuropathy on EMG, as seen here (both nerve conduction studies showed reduced motor and sensory action potential amplitudes). Currently considered vasculitis-related, existing reports (13 adult and 2 pediatric cases) show predominant motor involvement (moderate to severe), typically partial motor/sensory deficits, and poor clinical courses with partial treatment responses similar to this case [10-12]. In contrast to the pediatric cases reported by Vogelgesang et al. (1995), which evolved over weeks to months and showed better response to methotrexate, the clinical course of the present case was hyperacute (developing in days) and remained refractory. This suggests a more aggressive vasculitic process within the nerve-muscle unit. [12].

Three JDM evolution patterns have been identified: type 1 with mild/moderate initial activity and gradual recovery after one year (42%), type 2 with severe initial activity but faster recovery by one year (55%), and type 3 with the highest severity and very slow recovery (3%) [13]. Our patient corresponds to type 3.

Alternative treatment evaluation reveals severe cases should initiate high-dose methylprednisolone plus disease-modifying agent (methotrexate), reassessed every four weeks. Refractory cases warrant, sequentially, methylprednisolone with IVIG, azathioprine, cyclosporine, cyclophosphamide, mycophenolate, and/or biologics (rituximab, infliximab, or adalimumab) [4,7]. A Japan-led alternative proposes first-line azathioprine and cyclophosphamide (replacing methotrexate) [5]. Management followed the CARRA and SHARE guidelines [4,14]. However, biological therapies (such as rituximab), which are indicated for such type 3 (chronic-persistent) evolution patterns, could not be used due to socioeconomic barriers and lack of insurance coverage, representing a significant deviation from the ideal treatment algorithm.

## Conclusions

This case illustrates severe, refractory JDM with rare peripheral neuropathy, demonstrating early dysphagia, rapid functional decline, and persistent disability despite guideline-directed multimodal therapy (methylprednisolone pulses, methotrexate, IVIG, and azathioprine) over 48 months. Key clinical lessons include: (1) Early recognition of neurologic involvement as a high-risk feature. (2) Therapeutic challenges of rapidly progressive phenotypes. (3) Need for individualized intensification beyond standard protocols. These findings from a single case highlight gaps in managing atypical JDM presentations.
